# A dataset of stereoscopic images and ground-truth disparity mimicking human fixations in peripersonal space

**DOI:** 10.1038/sdata.2017.34

**Published:** 2017-03-28

**Authors:** Andrea Canessa, Agostino Gibaldi, Manuela Chessa, Marco Fato, Fabio Solari, Silvio P. Sabatini

**Affiliations:** 1 DIBRIS—University of Genoa, Genoa, GE 16145, Italy

**Keywords:** Visual system, Computer science, Biomedical engineering

## Abstract

Binocular stereopsis is the ability of a visual system, belonging to a live being or a machine, to interpret the different visual information deriving from two eyes/cameras for depth perception. From this perspective, the ground-truth information about three-dimensional visual space, which is hardly available, is an ideal tool both for evaluating human performance and for benchmarking machine vision algorithms. In the present work, we implemented a rendering methodology in which the camera pose mimics realistic eye pose for a fixating observer, thus including convergent eye geometry and cyclotorsion. The virtual environment we developed relies on highly accurate 3D virtual models, and its full controllability allows us to obtain the stereoscopic pairs together with the ground-truth depth and camera pose information. We thus created a stereoscopic dataset: *GENUA PESTO—GENoa hUman Active fixation database: PEripersonal space STereoscopic images and grOund truth disparity*. The dataset aims to provide a unified framework useful for a number of problems relevant to human and computer vision, from scene exploration and eye movement studies to 3D scene reconstruction.

## Background & Summary

Stereopsis is commonly dealt with as a static problem, because the disparity map obtained by a fixed-geometry stereo camera pair with parallel axes, is sufficient to reconstruct the 3D spatial layout of the observed scene. However, the capability of scanning the visual scene with the eyes, as for active vision systems, provides a number of contingent cues about the 3D layout of objects in a scene that could be used for planning and controlling goal-directed behaviors. Specifically, a purposeful approach can take into account attention mechanisms and a gaze-centric analysis of the scene. This is particularly true while interacting with peripersonal workspace, i.e., the space at short (reaching) distances, representing the next challenge of advanced cognitive robotic systems. A systematic collection of stereoscopic image pairs under vergent geometry, with ground-truth depth/disparity information, would thus be an ideal tool to characterize the problem of purposeful 3D vision. Nevertheless, these kinds of datasets are, unfortunately, rare or nearly absent.

Existing public datasets mainly provide scene images and range data: e.g., see 2.5D/3D Datase^1^, CIN 2D+3D^2^, Cornell RGB-D^3,4^, LIVE Color+3D^5,6^, B3DO^7^, Sun3D^8^. Among them, few provide stereoscopic images with disparity data: e.g., see Middelbury^9^, IMPART^10^, KITTI^11,12^, and SYNS^13^ datasets. Yet, they mainly follow a standard machine vision approach, i.e., with parallel optical axes for the two cameras (*off-axis* technique). In such a situation, vertical disparity is identically equal to zero over the whole field of view, thus reducing the search zone for stereo correspondence to horizontal lines, i.e., to a 1D problem. Moreover, horizontal disparity is proportional to the depth of the points in space.

A binocular visual system, belonging it to a human or a robotic system (e.g., see iCub^14^), is required to correctly fixate with the two eyes, i.e., to have both optical axes converging on the same point in space (*toe-in* technique). The resulting disparity patterns are considerably different from those derived by the *off-axis* technique^15^, and the search for stereo correspondence turns into a 2D problem. The horizontal disparity is close to zero at fixation, and assumes positive values for points closer than the fixation point and negative values for points farther away. Besides, vertical disparity takes on a small but significant value, providing a characteristic ‘cross’ shape.

**Figure 1:**
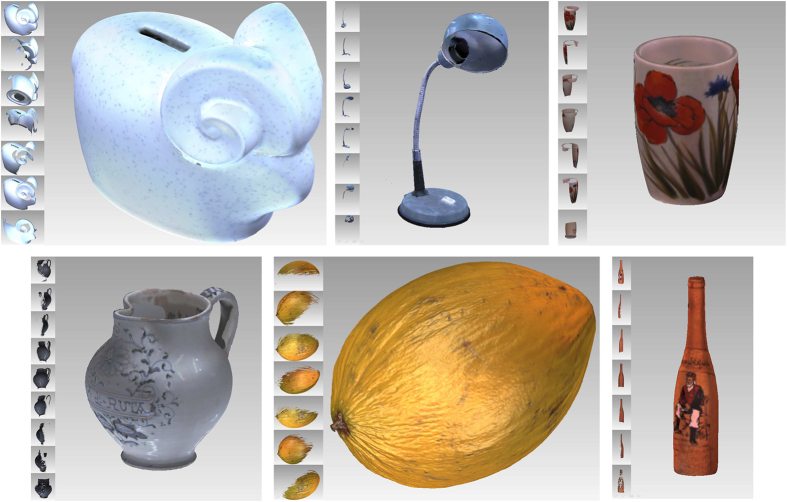
Examples of 3D model acquisition and registration. For each presented object, the insets on the left show the different 3D raw scans used to build the complete object model.

**Figure 2:**
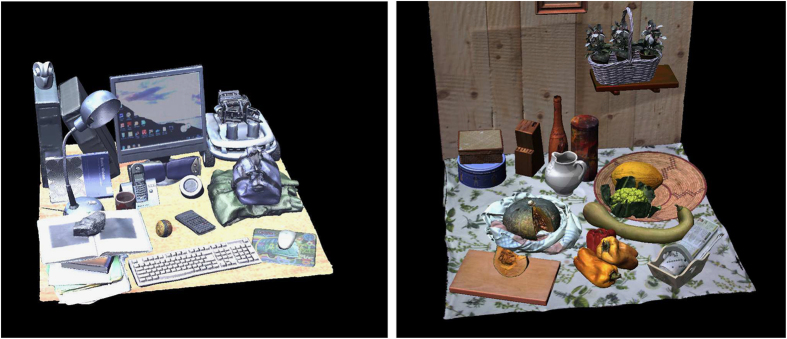


**Figure 3:**
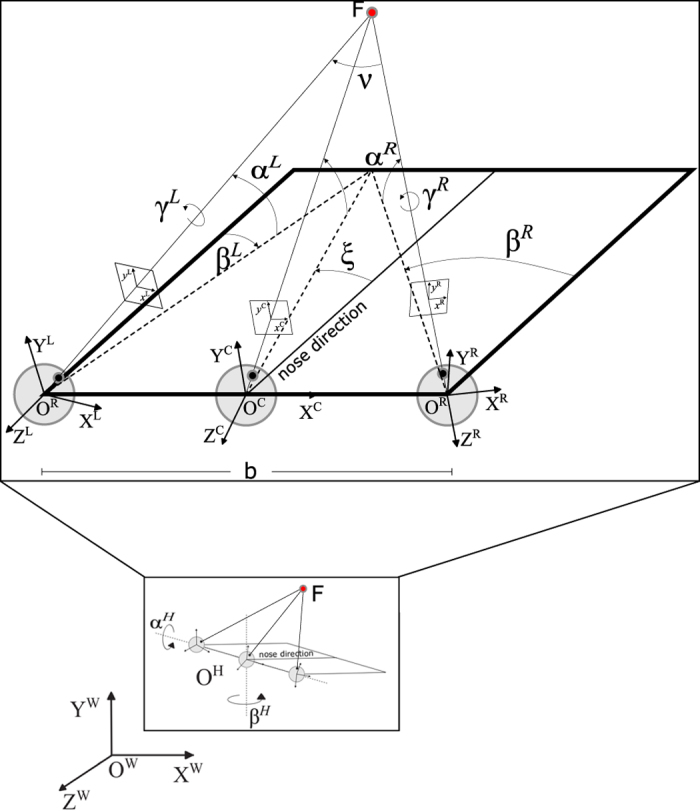
Schematic representation of the geometry of the binocular active vision system. *F* is the fixation point, *C* is the cyclopic position (halfway between the eyes), *L* and *R* are the left and right camera positions, separated by a baseline *b*=60 mm. The *α*, *β* and *γ* stand for the elevation (pitch), azimuth (yaw) and torsion (roll) angles of the left *L* and right *R* eye. The nose direction is the line orthogonal to the baseline and lying in a transverse plane passing through the eyes. The angles *ε* and *ν* stands for the binocular azimuth and vergence (see text for detailed explanation).

The dataset presented in this paper is meant to mimic the actual 6 degree-of-freedom eye pose of an active binocular visual system in peripersonal space, i.e., with camera pose exhibiting vergence and cyclotorsion as a function of the gaze direction^16^, (i.e., direction from camera to fixation point). Our approach relies on two complementary parts: 3D virtual models of natural scenes composed of in peripersonal space, which consist of accurate depth information and natural textures for the scenes (see Figs 1 and 2), and a graphic stereo vision simulator, to mimic the natural eye position of the human visual system (see Fig. 3).

The resulting dataset has a number of outstanding qualities, required for stereo benchmark^17^: (1) high spatial accuracy (≈0.1 mm), (2) realistic color texture with high resolution, (3) ground-truth disparity maps, (4) ground-truth occlusions and depth edges, (5) ground-truth position of stereo cameras, and (6) large number of stereo pairs.

Accordingly, this dataset has a large potential for use in different fields of research. In *Human Vision*, ground-truth data of natural scenes allow for a quantitative characterization of human behaviour, providing a useful means to create fully controlled and accurate visual stimulation for psychophysics and neuroscience experiments^18^. The stereo pairs can be used to investigate the influence of depth cue on: eye movement^19–24^, visual saliency and attention^19,25–32^, surface orientation perception^33–40^ and object recognition^41–45^. Considering recent widespread of 3D visualization methodologies, from low-cost TV monitors to head-mounted displays, the vergent geometry can be also useful to improve eye-hand coordination^46–48^, or for stereoscopic perception^49–51^ and image quality assessment^52–54^. The large number of stereo pairs can be used to collect retinal disparity statistics, for a direct comparison with the known binocular visual functionalities^55–62^. Specifically, the *ground-truth* quality of the data allows for an in-depth comparison that would not be possible otherwise^63^. Furthermore, the dataset can be used to learn monocular and binocular receptive fields^56,64–66^.

In *Machine Vision*, the ground-truth information included in the dataset, seldom provided in 3D databases^9^, is an optimal instrument to perform unconventional analyses of the problem, in order to develop and benchmark the algorithms themselves. The high accuracy and resolution of the ground-truth disparity data, makes the dataset optimal for an extensive evaluation of disparity estimation (for a recent review, see^67^). Specifically, since no databases of images with ground-truth *vector* disparity are available, the proposed database is unique in its kind. Exemplifying, it allows deriving quantitative performance indexes for horizontal and vertical disparity estimation algorithms, both on a pixel and a local basis^9,68–70^. Moreover, it can be used for image segmentation^71,72^ and object recognition^73–75^, surface orientation estimation^76,77^ and *Occlusion* and depth discontinuities estimation^17,71,77–84^, and visual saliency models^85–87^. The ground-truth position of the cameras can be used to benchmark stereo calibration^88^, epipolar geometry and camera pose estimation^80,89–95^.

The dataset can be also useful for algorithms not directly related to stereovision, like: structure from parallax^73,96^, three views geometry and trifocal tensor^97,98^, and multiple view geometry^97,99^, as well as feature matching^100^ and affine transformation^101^.

Summarizing, the present dataset of stereoscopic images provides a unified framework useful for many problems relevant to human and computer vision, from visual exploration and attention to eye movement studies, from perspective geometry to depth reconstruction.

## Methods

In order to render naturalistic stereoscopic images together with ground-truth disparity information, our methodology consists of two complementary parts. From the one side, it requires 3D virtual models of natural scenes, which provide both the natural texture of the objects composing the scene (see Fig. 2), and the accurate geometric information about the objects’ shape. Those models are required for the stereo vision simulator, in order to render naturalistic stereoscopic images accompanied by ground truth disparity information. Specifically, the simulator has been implemented to mimic the viewing posture of the human visual system in terms of vergent geometry and cyclotorsion. In this section, we describe the technical details about the 3D models acquisition and registration, and about the implementation of the stereo vision simulator.

### 3D model acquisition and composition

Aiming to study the peripersonal space, the scenes that we considered were bounded inside a workspace 1 m×1 m. The scenes were composed of real-world objects arranged in a cluttered way in order to have a high complexity structure. The scenes tried to replicate every-day life situations.

#### The 3D laser scanner

For the simulations shown in the following, we first captured 3D data from a real-world scene of the peripersonal space, by using a 3D laser scanner. To this purpose we selected a Konica Minolta Vivid 910, a fully portable sensor specifically designed for the acquisition of 3D images at close distance (in the range 0.6~2.5 m). The scanner combines a laser range-finder with a camera, providing digitized images with accurate distance as well as luminance information for every pixel in the scene.

The device emits a laser beam that scans the physical scene in equi-angular steps of elevation and azimuth relative to the center of the scanner. The distance of each point in the scene from the scanner is calculated by measuring the return time of the laser signal. The resulting range measurements generate a 3D representation of the scene, from the vantage point of the laser range finder. The device identifies the visible object surface at distances from 0.6 to 1.2 m, with an accuracy of 0.1 mm. The software provided by the vendor allows to acquire the same environment from different viewpoints, and to align and merge the different scans, in order to construct a full 3D representation of the scene. Each scan contained up to 307200 points acquired in 2.5 s. The device, providing three interchangeable lenses (focal distance: TELE 25 mm, MIDDLE 14 mm and WIDE 8 mm), allows for a variable angular field of view from ≈10 cm^2^ to ≈1 m^2^ (computed as the angular image area at near depth). Moreover, the device provides not just the point cloud, but also a polygonal mesh created with all connectivity information retained, thereby eliminating geometric ambiguities and improving detail capture. Furthermore, the scene camera also provides the color texture registered to the polygonal mesh, at a resolution of 640×480 pixels.

#### The acquisition of the single objects

Each object used for the scenes was first acquired separately, in order to obtain a full 360° model with no occlusions. The the laser scanner was used in TELE modality to obtain a high spatial resolution (approximately 30.000 3D points per cm^2^). Each object was scanned from different positions and then the scans were aligned and merged to obtain the full model. The number of scans for each object (≈20) varied according to the complexity and size of the object, and the position and orientation of the laser scanner is decided to reduce holes and occluded points in the scan data. The unconnected scans are aligned, first with a point-to-point manual procedure and then with an automated global registration procedure that minimizes the distance among the different scans. Finally, the merge procedure allows us to obtain the full connected model of the whole object. In general, a post-processing phase is required to obtain the final model. We perform hole filling or smoothing where required, using a custom region selection tool. The â€œreduce noise’ function smooths the data to reduce the acquisition noise, while preserving the shape and the original texture of the object. Then, the polygons that are not connected to the object are manually trimmed, and the ‘fill holes’ command is performed to ensure that no remaining holes are present in the model. The final model has a number of points ranging between 500.000 and 2.000.000, where each 3D point has its own color associated. Figure 1 shows some examples of the final object models.

#### The acquisition of the complete scenes

In order to recreate everyday living environments, we considered two cluttered scenes, an office table and a kitchen table (see Fig. 2). The real objects were mounted on a rotating table to facilitate the acquisition. The scenes were illuminated using a set of lamps at 6,500° K, to create diffuse illumination with white light, so to obtain texture objects as much similar as possible to the real one.

We proceeded following a simple protocol to generate the final scene models. First of all, we take 8 scans of the entire scenes with the scanner in WIDE modality, rotating the table by step of 45°, thus obtaining a full 360° model. The scans were then aligned (manual and automated registration procedure) and then merged, as for the single objects. The obtained raw model is characterized by a lower spatial and texture resolution with respect to the single object models, and by a large number of holes, due to the high complexity of the scene, where objects occlude each other.

After these steps, each single object model was aligned within the raw scene, with a procedure similar to the one used to align the different scans of a single object. Each object was first aligned within the full scene with a point-to-point manual procedure. The alignment was then refined using an automated global registration procedure that minimizes the distance between the high resolution object and its low resolution version in the full scene. Finally, the points belonging to the low resolution were manually selected and removed from the scene, recreating it as a composition of high resolution and high precision object models. The scene was then aligned with a right hand world coordinate system with metric unit, where the *x* axis is directed from left to right, the *y* axis is directed from down to up, perpendicular to the table surface, and the *z* axis points toward the viewer. The origin of the three axes of the coordinate system was located in the center of the table surface.

The scene, was exported as a clean VRML file. It is worth noting that, in this file format, the single objects are separated, allowing us to set specific material properties for each object.

The presented methodology was used to compose the two virtual environments, as in Fig. 2.

### Stereo vision simulator of images and ground-truth data

The simulator generates the sequence of stereo image pairs, the depth maps with respect to both the cyclopic position and the left camera, and the exact pose of both the left and the right camera, by considering a binocular head moving in the 3D space and fixating a 3D point (*X*^*F*^, *Y*^*F*^, *Z*^*F*^). The geometry of the system is shown in Fig. 3.

Once the initial position of the head is fixed, then different behaviours are possible:

- to move the eyes by keeping the head (position and orientation) fixed;
- to change the head orientation, thus mimicking neck movements;
- to change both the head orientation and position, thus generating more complex motion patterns.

#### Head movement

The head is characterized by the following parameters (each expressed with respect to the world reference frame (*X*^*W*^, *Y*^*W*^, *Z*^*W*^)):

- head position **O**^**H**^;
- nose direction $n=n(\alpha^{H},\beta^{H})$, function of the elevation $\alpha^{H}$ and azimuth $\beta^{H}$ angles of the neck;
- fixation point **F**=(*X*^*F*^, *Y*^*F*^, *Z*^*F*^).

Specifically, the nose direction is the line orthogonal to the baseline and lying in a transverse plane passing through the eyes. Note that the rotation of a rigid body, generally expressed by yaw, pitch and roll for rotations about the *X*, *Y* and *Z* axis, respectively, is here expressed as azimuth (yaw), elevation (pitch) and torsion (roll), to maintain a notation more familiar to eye movements studies. Note that the elevation (pitch), azimuth (yaw) and torsion (roll) define a rotation about the *X*, *Y* and *Z* axis, respectively. For the head, the elevational and azimuthal orientation are described by the following rotation matrices:

(1)RαH=[1000cosαL/R−sinαL/R0sinαL/RcosαL/R]RβH=[cosβL/R0sinβL/R010−sinβL/R0cosβL/R]

The complete rotation of the head is defined by composing in cascade the above rotations following a Fick gimbal system^102,103^:

(2)RH=RβHRαH

Since $R^{H}$ is composed of rotations about the horizontal and vertical axes, only, the resulting head pose is characterized by no torsional component, according to the kinematics of the Fick gimbal system. From this, it is possible to write the pose matrix for the head as:

(3)PWH=[RHOH01]

#### Toe-in technique to simulate eye movements

**Figure 4:**
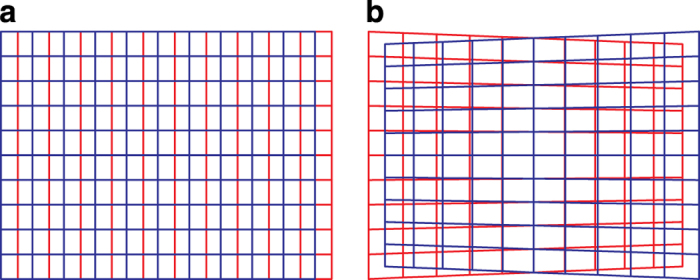
The projections of a fronto-parallel rectangle onto the image planes, drawn in red for the left image and blue for the right. The texture applied to the rectangle is a regular grid. (**a**) The projection obtained with the off-axis technique: only horizontal disparity is introduced. (**b**) The projection obtained with the toe-in technique: both vertical and horizontal disparities are introduced.

**Figure 5:**
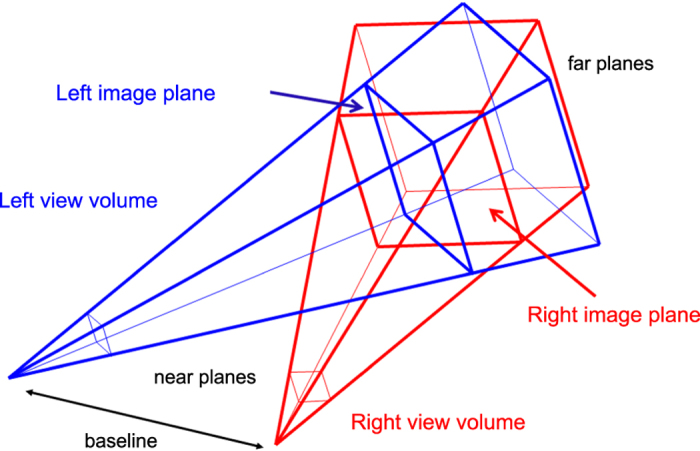


As anticipated, to render stereoscopic stimuli on a screen, two techniques exist: the *off-axis* and the *toe-in* techniques. The first one is used to obtain stereo 3D for a human observer, as for the cinema and television stereo 3D^104^. The disparity patterns produced by the off-axis and toe-in techniques, when observing a frontoparallel plane, are shown in Fig. 4a,b, respectively. In this context, the correct way to create the stereo pairs is the toe-in method: the projection direction of each camera is set to the target point (the fixation point **F**) through a proper rotation. Then the left and the right views project onto two different planes, (see Fig. 5). The cameras are characterized by the following parameters (each expressed with respect to the head reference frame):

- camera position **O**^*L/R/C*^;
- camera orientation $R^{L/R/C}=R^{L/R/C}(\alpha^{L/R/C},\beta^{L/R/C})$, function of the elevation *α* and azimuth β angles;

Moreover, the cameras have pinhole optics with unitary focal length. The origin of the left and the right view volume is fixed at

(4)TL/R=[±b200]T

while the cyclopic view volume is located at the origin of the head reference frame. The elevational and azimuthal orientation of the cameras are described by the rotation matrices in equation (2). To emulate the behavior of a couple of verging pan-tilt cameras the complete rotation of each camera is defined composing in cascade the above rotations following an Helmholtz gimbal system^103^:

(5)RL/R/C=RαL/R/CRβL/R/C

In this way, it is possible to insert a camera in the scene (e.g., a perspective camera), to obtain a stereoscopic representation with convergent axes and to decide the location of the fixation point.

#### Binocular coordination of eye/camera movements

A single eye/camera, like any rigid body, has three rotational degrees of freedom. Though, in a head centered reference frame, only two angles are sufficient to determine the gaze direction: namely the azimuth (yaw) and the elevation (pitch) of the target, as by equation (5). This implies that the eye/camera could, in principle, assume an infinite number of torsional poses for any gaze direction, while correctly fixating a given target.

Considering a human observer, the complete 3D pose of a single eye is defined (limited) by the Listing’s law (LL), which specifies the amount of the torsional angle with respect to the gaze direction^105^. The ecological reason subtending LL is to provide a *motor advantage* to the visual system, through an optimization of the oculomotor control. In fact, according to LL, each eye would move always rotating along the shortest geodetic path from the primary position (i.e., straight ahead gaze direction).

The situation differs if we consider a binocular visual system, because a thoughtful motor coordination of the two eyes/cameras has a meaningful perceptual significance. The relative torsion of the two eyes, defined as the cyclotorsion, has in fact the goal of reducing the search zone for retinal correspondence^106–108^, thus facilitating the problem of stereopsis^109^. Empirical evidences showed how the kinematics of the eyes slightly deviated from LL. To obtain perceptual advantages in binocular coordination, the torsional posture of each eye changes with eye convergence, particularly in close viewing^16,110–115^. This difference is defined as the binocular extension of Listing’s Law (L2)^16,111,115,116^. The resulting posture provides an alignment of the horizontal meridians of the left and right eyes. This alignment reduces the search zone for retinal correspondence, thus providing a *perceptual optimization* of eye pose for stereopsis^106–108^.

During convergence movements, once defined the starting vergence angle, the eyes’ rotation axes remain confined to planes that rotate temporally and roughly symmetrically by $\phi_{l}$ and $\phi_{r}$ angle, for the left and the right eye, respectively. These convergence dependent changes of torsional positions (i.e., orientation of Listing’s plane) have been referred to as the binocular extension of LL or, in brief, L2 (ref. 117). The grater is the convergence, the more the planes rotate temporally, implying that during convergence, there is a relative excyclotorsion on upgaze, and a relative incyclotorsion on downgaze. To mimic the natural eye pose, taking into account the tilting of the Listing’s plane, we had to consider a torsion of the camera $\gamma^{L/R}$ given by^118^:

(6)tanγL/R2=−tanαL/R2[tanϕL/R+tanβL/R1+tanϕL/RtanβL/R]

with

(7)ϕL=−ϕR=δ2arcsinsinν2cosξ2

where $\nu =\beta^{R}-\beta^{L}$ and $\xi =\frac{\beta^{L}+\beta^{R}}{2}$ are the vergence and azimuth angles, respectively. The parameter *δ* controls the balance between the motor advantage provided by LL, and the perceptual optimization for stereopsis, provided by L2. In all the following simulations we decided to follow previous literature and to adopt *δ*=0.8 (refs 62,63,108,114). Thus, the angle *γ* provide the amount of torsion (roll) to be applied to the left and the right cameras for a given azimuth and vergence distance, in order to obtain a camera pose compliant with the L2 law.

For taking into account the torsion *γ* of left and right cameras we had to pre-multiply the rotation matrices $R^{L/R}$ by a torsional rotation matrix:

(8)RγL/R=[cosγL/R−sinγL/R0sinγL/RcosγL/R0001]

The complete rotation of the view-volumes is described by the following relation:

(9)RL2L/R=RL/RRγL/R

where the subscript *L*2 is used to indicate that it is Listing’s law compliant matrix.

It is now possible to obtain the pose matrix for the cameras in head reference frame:

(10)PHL/R/C=[RL2L/R/COL/R/C01]

and to compose the matrices to obtain the pose matrix in world reference frame:

(11)PWL/R/C=PWHPHL/R/C

#### Rendering of stereo pairs and ground-truth data

The scene is rendered in an on-screen OpenGL context. The available SoOffScreenRenderer class is used for rendering scenes in off-screen buffers and to save to disk the sequence of stereo pairs.

The renderer engine allows us to produce stereo images of different resolution and acquired by cameras with different field of views. In particular, the following ‘optical’ parameters can be set:

- resolution of the cameras (the maximum possible resolution depends on texture resolution and on the 3D point cloud density);
- horizontal and vertical field of view (HFOV and VFOV, respectively);
- distance from camera position to the near clipping plane in the camera’s view volume, also referred to as a viewing frustum, (nearDistance);
- distance from camera position to the far clipping plane in the camera’s view volume (farDistance);
- distance from camera position to the point of focus (focalDistance).

To compute the ground-truth data it is necessary to exploit the resources available from the graphics engine by combining them through the computer vision relationships that describe the geometry of two views, typically used to obtain a 3D reconstruction.

Formally, by considering two static views, the two camera reference frames are related by a rigid body transformation described by the rotation matrix $R_{L2}^{L/R}$ and the translation $T=T^{R}-T^{L}$. The x^*L*^ homogeneous coordinates on the left image plane are back-projected on the 3D scene, and the obtained 3D points are then re-projected on the right image plane, obtaining the associated x^*R*^ homogeneous coordinates by perspective division, in the following way^119^:

(12)λRxR=RL2R[(RL2L)−1λLxL−T]

where $\lambda^{L}$ and $\lambda^{R}$ are the depth values.

To apply the relationship described in equation (12), we first read the depth map (*w*) of the camera through a specific method added in the SoOffScreenRenderer class, then we obtain the depth values with respect to the reference frame of the camera in the following way:

(13)λ=fnw(f−n)−f

where *f* and *n* represent the values of the far and the near planes of the virtual camera, respectively.

The simulator provides:

- the images saved from the off-screen buffers, for the left, right and cyclopic postions.
- the depth values, computed from the depth map by following equation (13), for the cyclopic position and for the left camera.

Though in general the disparity map of a stereo pair would be calculated with respect to one of the two image planes (left or right image)^9^, to avoid asymmetry problems we decided to refer also to the image plane of a cyclopic camera^120^, located in the mid point between the left and right cameras, pointing along the gaze line at the selected fixation point. Given the projection of a 3D virtual point on the cyclopic image plane, the disparity maps were computed by the correspondent projections in the left and right image planes. Starting from the image coordinate x^*C*^ of the cyclopic image and the depth values $\lambda^{C}$ obtained by the depth map, the image coordinate x^*L*^ and x^*R*^ of the left and right view are obtained in the following way:

(14)λLxL=RL2L[(RC)−1λCxC−TL]λRxR=RL2R[(RC)−1λCxC−TR].

By exploiting the relationship of equation (12) and equation (14), given the knowledge of the depth values for a camera, and of the relative pose of the two cameras, we compute the image coordinates for both the views, and then we define the horizontal *d*_*x*_=*x*^*R*^−*x*^*L*^ and the vertical *d*_*y*_=*y*^*R*^−*y*^*L*^ disparities.

Due to the different position of the left and right cameras, some points in one image may happen not to be visible on the other image, depending on the 3D structure of the scene. Those points are defined as *occlusions*, and can be computed by the ground-truth disparity map, since the forward-mapped disparity would land at a location with a larger (nearer) disparity. Similarly, the ground-truth disparity can be used to compute the depth discontinuity regions, i.e., those regions whose neighboring disparities differ by more than a defined threshold. On this basis, we computed two binary maps, one for the occluded points and one for the depth discontinuities.

### 3D fixation database

Once we obtained the VRML models of our 3D environment, we need to model a visual system able to explore this virtual environment, making fixation movements on the objects’ surfaces.

**Figure 6:**
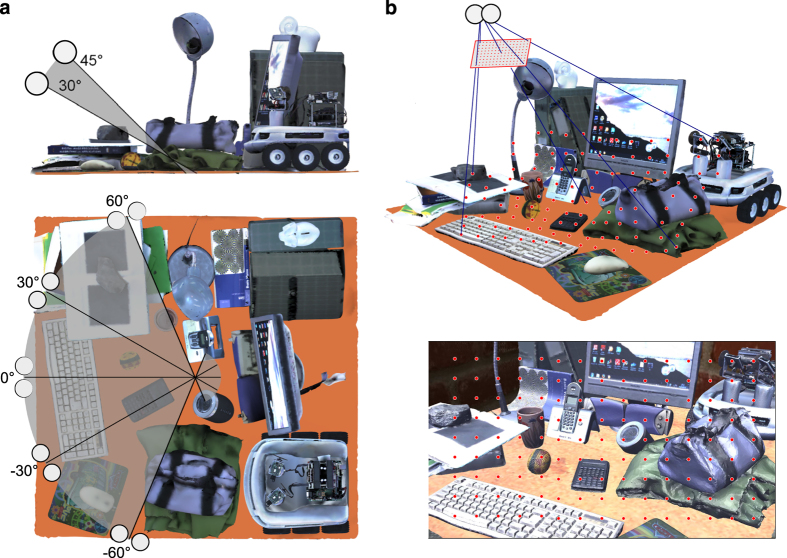
Representation of head position and 3D fixation point in the virtual scene. (**a**) Side and top view of the position of the 10 vantage points used to place the head within the 3D scene. The solid thick lines represent the nose direction for each head position. (**b**) Image acquired by the cyclopean camera from one of the ten vantage points (top), and geometrical configuration of the camera system with respect to the 3D scene (bottom). The red dots represent the 9×15 grid of points equally spaced on the image plane of the cyclopean camera (top), that are used to compute the actual 3D fixation points in the scene (bottom). The black solid line represents the nose direction, while the green lines represent the four most lateral fixations within the grid of fixation points.

For the two virtual worlds we considered 10 different vantage points for the subject head, corresponding to different positions and orientations of the nose direction of the head (see Fig. 6a). Facing the scene, the head was put with the nose direction pointing to its center. The head position orbited uniformly around center of the scene with an azimuth angle in the range [−60° 60°] by steps of 30° and 2 elevation angles of 30° and 45°. In each of the vantage points, the head was then put at a fixed vergence distance of ≈3° (=155 cm) from the closest object along the nose direction. The distance between the nodal points of the left and right, i.e., the baseline, is 60 mm. While stereoscopic algorithms are generally insensitive to the baseline, which works as a *scaling* factor, this baseline has been selected to be close to the interpupillary distance of a human being^121^. The fixation points were obtained by means of the cyclopic virtual camera centered in between the left and right cameras, and oriented along the same gaze line direction. A grid of 9×15 equally spaced image points was projected on the 3D scene (see Fig. 6b). The 3D coordinates of the fixation point in the scene were thus computed as the intersection between the binocular line of sight and the closest visible surface of the scene, i.e., the closest triangle of the model’s mesh. The procedure was repeated for the two virtual scenes considered. The proposed approach is intended to provide an even and complete sampling of the visual space, thus we considered uniform spacing for both head position and gaze direction. Nevertheless, it is worth considering that human fixations are neither evenly nor randomly distributed within the environment. Generally, human fixation strategy is preferentially directed towards behaviorally significant points^122^. Specifically in a 3D scene, the distribution of vergence angle is biased towards closer points with respect to the possible fixations within the visual scene^62^, because close targets more likely and more immediately attract our gaze^20,22,23^.

Active fixations on the scanned 3D scenes were simulated by accessing to the image textures and to the depth map. The depth ranged from ≈500 mm to ≈2,200 mm. For each of the 2×10×9×15 given camera poses we obtained the left and right retinal images and the horizontal and vertical cyclopic disparity maps. To eliminate bias due to the disposition of the objects in the scenes, we decided also to calculate, for each fixation, the mirrored cyclopic disparity maps. Mirrored disparity maps were always obtained from equation (14) mirroring the depth $\lambda^{C}$ along the middle vertical line. Accordingly, we obtained a dataset of 5,400 binocular fixations, constituted by the left and right views and the associated disparity patterns.

### Code availability

Together with the present dataset, the necessary code for its proper use will be made available at http://www.pspc.unige.it/Code/index.html, but can be also downloaded at https://sourceforge.net/projects/genua-pesto-usage-code/. The Matlab code has been developed on R2011a version and is compatible with all the subsequent versions. The C/C++ code has been developed in Unix environment and has been tested with Windows OS (Microsoft Visual Studio 2010). This code requires the *libpng* library (http://www.libpng.org) in order to load the *png* images of the database.

- *Data loading* (Matlab and C/C++): correct loading of images, depth maps and head/eye position;
- *Disparity computation* (Matlab and C/C++): computation of binocular disparity form the depth map;
- *Occlusion computation* (Matlab): computation of the ground-truth occlusion map from the disparity map;
- *Depth edges computation* (Matlab): computation of depth edges from disparity map;
- *Validation indexes computation* (Matlab): compute the indexes for validation and testing of disparity algorithm. The horizontal and vertical disparity estimation indexes are computed as the mean absolute error and standard deviation, with respect to the ground-truth disparity^9^. The image-based indexes are the MAE, NCC and SSI, described in the Technical Validation section.

## Data Records

The database is available at Dryad Digital Repository (see Data Citation 1). We divided it in two sub-datasets, one for each of the two virtual worlds (see Fig. 2). Each sub-dataset is firstly divided according to the 10 different vantage points for the head. These are organized and stored in separate zip files using two numeric indexes, according to the following file name format: #scn HP #a #e, where:

- #scn defines the virtual world, K for the kitchen and O for the office,
- HP stands for *head position* (head azimuth and elevation),
- #a the head azimuth index, which is an integer from −2 to 2 that corresponds to angles in the range[−60°, 60°], with step of 30°,
- #e the head elevation index, which can assume value 1 and 2 corresponding to angles of 30° and 45°, respectively.

Within the above folders, for each of the 9×15 cyclopean image points the following data are stored:

- Stereo-pair images (left and right camera images) as PNG files (1,921×1,081 pixels).
- Cyclopic images as PNG files (1,921×1,081 pixels).
- Cyclopic camera depth map as PNG files (1,921×1,081 pixels).
- Left camera depth map as PNG files (1,921×1,081 pixels).
- Info file is provided in TXT format with all the geometrical information regarding the virtual stereo head for the actual fixation, i.e., the head position, head target and head orientation (world reference frame); the camera position and camera orientation (both world and head reference frame) for the left cyclopean and right cameras; the binocular gaze direction, expressed as version elevation and vergence (head reference frame), or as quaternion (both world and head reference frame). The file also contains the normalization values for the conversion of the depth map from PNG format to real depth value in mm.

**Figure 7:**
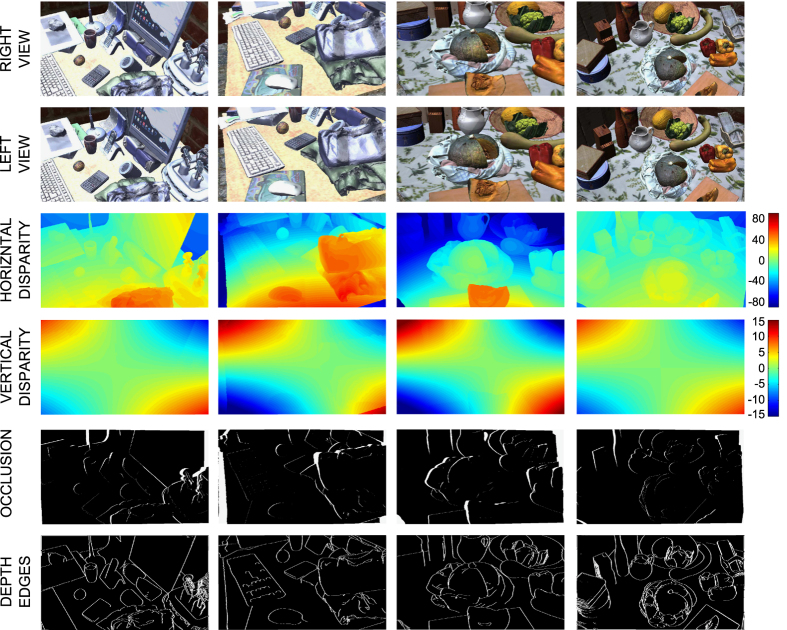
Example of stereoscopic pairs from the dataset, including, from top to bottom, the left and right views, the horizontal and vertical ground-truth disparity maps, and the occlusion and edge maps. In the disparity maps, reported in pixel, hot colors represent crossed horizontal disparity and right-hyper vertical disparity, whereas blue colors represent uncrossed horizontal disparities and left-hyper vertical disparities, according to the colorbars on the right.

The file names are defined according to the following format:

XX_HP_#a_#e_H_#h_V_#v;

where XX denotes the data name (e.g., *cycdepth* for the cyclopic depth map), HP_#a_#e recalls the information about the head position, as for the folder name. The indexes #h and #v describe the azimuth (from −7 to 7) and elevation (from −4 to 4) of the binocular fixation point within the 9×15 grid of gaze directions. The database is accompanied by two functions. The first, *Disparity_computation*, available both in Matlab and C++, takes as arguments a.txt info file and the associated cyclopic and left depth map PNG images and returns the following data (see Fig. 7):

- horizontal and vertical cyclopic disparity, stored in two separated binary files of 1,921×1,081 floating point values.
- horizontal and vertical left disparity, stored in two separated binary files of 1,921×1,081 floating point values.
- Cyclopic camera depth map in mm, stored in a binary file of 1,921×1,081 floating point values.
- Left camera depth map in mm, stored in a binary files of 1,921×1,081 floating point values.

The function, *compute_occlusion*, available in Matlab, takes as arguments the stereo pair and the associated horizontal and vertical ground-truth disparity, and returns the ground-truth occlusion mask for the actual stereo-pair, stored as a binary image, and the right image of the stereo pair, warped by the ground-truth disparity and removed the occluded pixels (see Fig. 7) The function, *compute_depth_edges* takes as arguments the horizontal and vertical ground-truth disparity maps and the depth maps, and computed the and depth edge map, stored as a binary image.

## Technical Validation

In order to provide a technical validation of the released dataset, the two complementary parts that constitute the approach have to be considered separately: the 3D virtual models and the VR simulator. The former has to be tested with respect to the accuracy of the 3D models obtained, whereas for the latter we will provide a validation of the correctness of the geometrical projection and rendering of the stereoscopic pair.

### 3D virtual models: acquisition and registration error

On each single scan, the manufacturer of the used laser scanner guarantees a root mean square error of 1 mm. The post-processing procedure allows us to resolve every possible problem in the different scans, due to unwanted surface reflection and to acquisition noise. The manual alignment and automated registration of the different scans might introduce a larger error, if not performed accurately.

Table 1 reports the maximum, average and standard deviation of the registration error in mm, measured on six objects randomly selected among all the scanned objects (see Fig. 1), and the mean values computed over all the object models used to assemble the scenes. While few 3D points with notable error might remain in the final model, the low average error and standard deviation provide a measurement of the quality of the obtained object models. In fact, the use of the TELE modality for the acquisition of the single scan, combined with a proper post-processing procedure allows us to obtain a spatial accuracy that is comparable to the accuracy of the device.

### VR simulator: disparity reconstruction error

The obtained virtual worlds are used within the virtual simulator to generate the stereoscopic pairs as well as the depth and ground-truth disparity maps. The disparity map can be interpreted as the transformation to obtain, from a pixel on the left image, the corresponding pixel on the right image. From this perspective, in order to assess the correctness of our approach, it is possible to use the image quality indexes that are commonly used to evaluate the errors between a distorted image and a reference image (see^69^, as review).

In stereo vision, two common approaches are used to evaluate the performance of disparity estimation algorithms (see^9^), a disparity-based and an image-based approach. The former relies on a ground-truth knowledge of the disparity, and computes indexes like the mean absolute error or the standard deviation with respect to the estimated map. If the ground truth disparity is not available, it is possible to warp the right image by the binocular disparity, in order to ‘reconstruct’ the left image, and compute indexes of similarity between the left (original) and right (warped) images.

Considering that our methodology provides the ground-truth disparity, if the geometrical projection and the rendering engine are correct, it should be possible to effectively reconstruct the left image by the right one, with negligible error. We thus used a bilinear interpolation algorithm, and we evaluated the obtained results over the whole dataset, using three error parameters sensitive to different image features:

- the mean absolute error (MAE)^9,70^ is computed at pixel level, by averaging the absolute intensity differences of original and reconstructed images,
- the normalized cross correlation (NCC)^9^, being the 2D version of the Pearson product-moment correlation coefficient, it provides a similarity metric that is invariant to changes in location and scale in the two images,
- the structure similarity index (SSIM) has been conceived to mimic the visual quality assessment methods of the human visual system, and is insensitive also to local luminance and contrast variations between the two images^68,69^.

**Figure 8:**
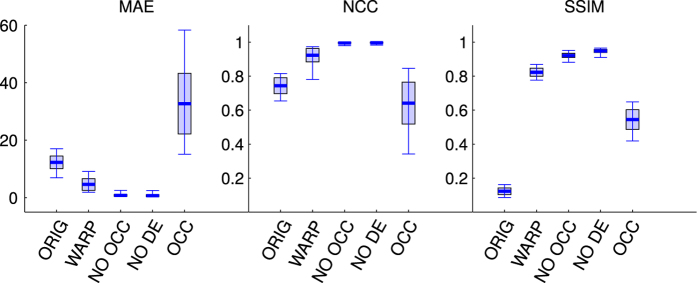
Disparity reconstruction error, computed on three different stereo quality indexes over the whole dataset, and represented as median value (horizontal thick line), first and third quartile (rectangle) and range (whiskers). Three different indexes are represented, from left to right: the Mean Absolute Error (MAE^9,70^), the Normalized Cross Correlation (NCC^9^) and the Structure Similarity Index (SSIM^68,69^). Each index has been computed for the original stereo pair (ORIG), not considering the occluded areas (NO OCC), not considering both the occluded areas and the depth edges (NO DE), and finally considering only the pixels corresponding to the occluded areas and the depth edges (OCC).

Figure 8 shows the median (solid thick line) of the three indexes computed over the whole stereo pair dataset, together with the first and third quartile (box), and the range (whiskers). Although the images have been rendered in color (see Fig. 7), the indexes have been computed on the gray level images (from 0 to 255), for the sake of simplicity. The indexes were first computed on the original stereo pair (ORIG), in order to obtain a reference value, and between the left original image and the warped right image (WARP). It is clearly evident how the warping reduces the absolute error and increases the correlation and the structure similarity between the pairs. In order further to validate the approach, it is worth considering that in stereoscopic vision some region on one image are occluded in the other one, and should be removed from the computation^9^. From this perspective, also the depth edges should not be considered since the image might suffer of rendering problem alongside the edges^9^. Hence, the computation was also performed on three different image regions over the warped pair: not considering the occluded areas (NO OCC), not considering both the occluded areas and the depth edges (NO DE), and finally considering only the pixels corresponding to the occluded areas and the depth edges (OCC). Since occluded regions and depth edges reasonably suffer of large errors, removing them provides a marked positive effect on the reconstructed stereo pair. In fact, the MAE is considerably reduced, and both the NCC and SSIM tend to their maximum. The removal of depth edges results in a further improvement, providing reconstructed images with a negligible error (<0.7 gray levels), high correlation (>0.997) and large structure similarity (>0.95).

Summarizing, the proposed methodology allows us to: (1) obtain 3D geometric models of real scenes with high spatial fidelity, and (2) render realistic stereoscopic pairs with ground-truth disparity characterized by accurate perspective geometry, thus assessing the correctness of the released dataset and validating the approach.

## Additional Information

**How to cite this article:** Canessa, A. *et al.* A dataset of stereoscopic images and ground-truth disparity mimicking human fixations in peripersonal space. *Sci. Data* 4:170034 doi: 10.1038/sdata.2017.34 (2017).

**Publisher’s note:** Springer Nature remains neutral with regard to jurisdictional claims in published maps and institutional affiliations.

## Supplementary Material



## Figures and Tables

**Table 1:** Table summarizing registration errors, expressed in mm, on single 3D object models.

	**Moneybox**	**Lamp**	**Pentray**	**Jug**	**Melon**	**Bottle**	**Mean**
Maximum	2.971	6.599	1.260	2.757	2.654	4.030	3.578
Average	0.066	0.100	0.051	0.085	0.084	0.110	0.112
Std. Dev.	0.103	0.176	0.054	0.128	0.098	0.128	0.124
The table reports the maximum, average and standard deviation of the error after the global registration procedure, for six 3D object models (see Fig. 1), randomly selected among those used to construct the virtual worlds. The last column reports the mean values over all object models.							
